# Ensuring guideline adherence and cost savings in stress ulcer prophylaxis practices in the intensive care unit: a pre-post education study

**DOI:** 10.3389/jpps.2025.14067

**Published:** 2025-03-04

**Authors:** Yunus Emre Ayhan, Namigar Turgut

**Affiliations:** ^1^ Prof. Dr. Cemil Taşcıoğlu City Hospital, Department of Clinical Pharmacy, Istanbul, Türkiye; ^2^ Department of Anaesthesiology and Reanimation, Prof. Dr. Cemil Taşcıoğlu City Hospital, University of Health Sciences, Istanbul, Türkiye

**Keywords:** stress ulcer prophylaxis, proton pump inhibitor, intensive care unit, clinical pharmacist, cost-saving

## Abstract

**Objective:**

This study aims to enhance adherence to the guideline through an educational program focused on reducing inappropriate use of stress ulcer prophylaxis (SUP) and cost savings in the intensive care unit (ICU).

**Method:**

This study was designed as a nonrandomized, controlled, prospective study created according to the pre-education (PreEd) and post-education (PostEd) evaluation model and conducted between January and July 2024. The appropriateness of SUP uses for the indication was evaluated according to the Sociedade Portuguesa de Cuidados (SPC) SUP guideline. Adherence rates to the SPC SUP guideline and the costs associated with nonadherence were evaluated.

**Results:**

495 patients were included in the study, 244 in PreEd and 251 in PostEd. 58.2% of the patients were male, and the hospitalization was mainly for medical reasons (59.6%). The mean ± SD rate of patients with appropriate SUP indication was 38.3 ± 41.6% in PreEd and 47.8 ± 42.8% in PostEd (p = 0.005). The total costs of inappropriate indication and proton pump inhibitor use in PreEd and PostEd were 272 dollars and 246 dollars, respectively (p = 0.007). Accordingly, when inappropriate SUP agent use was calculated per patient in both periods, the total cost saving was 34 dollars.

**Conclusion:**

Inappropriate SUP use is common in the ICU. Adequate adherence to guidelines and proactive involvement of clinical pharmacists may reduce inappropriate SUP use and associated costs.

## Introduction

Intensive care unit (ICU) patients are prone to developing stress-related gastrointestinal (GI) bleeding, which is associated with increased morbidity and mortality. Respiratory failure, hypotension, coagulopathy, and especially prolonged mechanical ventilation (MV) are the most critical clinically significant risk factors for GI bleeding in patients [1, 2]. Many studies have shown that invasive MV for 48 h or longer and coagulopathy are two independent risk factors for clinically significant upper GI bleeding in ICU patients [3, 4].

Stress ulcer prophylaxis (SUP) is widely practiced in ICUs worldwide and is often (up to 70%) used inappropriately [5–7]. Proton pump inhibitors (PPI) are among the most commonly used medications in critically ill patients for SUP. However, the inappropriate and quite inconsistent use of PPIs in ICUs has added unnecessary costs, enhanced risks related to adverse drug reactions, and possible complications like pneumonia, *Clostiridioides difficile* infections, hypomagnesemia, and bone fractures [5].

A few studies have assessed adherence with SUP guidelines and institutional standards under the surveillance of a pharmacist. The results of these studies implied that pharmacist supervision reduced the inappropriate use of SUP in patients and its associated healthcare costs [5–11]. One of these studies noted that the intervention and adjustment of pharmacists reduced the incidence of inappropriate use of SUP and its associated costs from $26.75 and $2433 per 100 patient days pre-intervention to $7.14 and $239.80 per 100 patient days post-intervention with p < 0.001. The same study emphasized that a comprehensive multidisciplinary approach must be implemented to decrease inappropriate SUP use in the ICU [8].

The aim of this study is to enhance adherence to the guidelines through an educational program focused on reducing inappropriate use of SUP in the ICU. The goal is to promote appropriate use of SUP based on indications, leading to cost savings.

## Materials and methods

### Study design and patients

This study was designed as a non-randomized, controlled, prospective study using a pre-post education evaluation model. It was conducted in the anesthesia and reanimation ICU of a training and research hospital in Türkiye, between January 2024 and July 2024 (6 months). The study was conducted in pre-education (PreEd) and post-education (PostEd). In PreEd, the SUP use of patients in the ICU was observed observationally for 3 months (1 January 2024–1 April 2024). In the study, the SUP education program in the ICU was implemented for ICU physicians after examining the patient data in the first three months of the study. In the 3-month PostEd after the education program (3 April 2024–3 July 2024), SUP was only used in patients in the ICU, and no intervention was observed. Throughout the study period, the appropriateness of SUP uses for the indication was evaluated according to the Sociedade Portuguesa de Cuidados (SPC) SUP guideline [12].

### Inclusion and exclusion criteria

Patients aged ≥18 years, those with an ICU stay longer than 24 h, and those using PPIs for SUP were included in the study. Patients with a diagnosis of gastric cancer, history of GI, those with subtotal/total gastrectomy, those using PPIs for treatment indications such as dual antiplatelet therapy, and those admitted to the ICU with GI bleeding were excluded from the study.

### Data collection

Sociodemographic information, disease and medication history, existing laboratory values (coagulation parameters, procalcitonin, c-reactive protein, etc.), culture results, MV status, nutritional status, GI system bleeding status, pneumonia status, presence of *Clostiridioides difficile*, appropriate/inappropriate SUP use days and costs were obtained from the patient’s treatment file and the hospital information management system with the utmost respect for personal privacy conditions.

### Education program

The education program was organized on 3 April 2024, after completing the 3-month PreEd review. The 1-hour education program was presented face-to-face to ICU physicians by a clinical pharmacist and an intensive care specialist physician. Eight physicians attended the educational program, including attending 4 physicians and 4 residents, all of whom had prescribing authority in the ICU. The content of the education included education on SUP, pathophysiology of stress ulcer, SUP risk/benefit situations, guidelines for SUP, introduction of appropriate SUP criteria according to the SPC SUP guideline, and frequently inappropriate SUP prescription situations in ICU, and the correct time to stop SUP. The evaluations obtained during the PreEd review in the ICU were also presented to the physicians participating in the education.

### Assessment of the stress ulcer prophylaxis use

SUP use of ICU patients was evaluated throughout the week. SUP practices of ICU patients and patient data were reviewed by a clinical pharmacist and an intensive care specialist and evaluated for compliance with the SPC SUP criteria in terms of indication [12]. The use of appropriate SUP was determined based on the presence of either one major risk factor or two minor risk factors. Patients who met the criteria for either of these groups were considered for appropriate PPI use for SUP.

Major Risk Factors for SUP:

• Coagulopathy: Platelet count <50,000/m^3^, an INR superior to 1.5, or a aPTT superior to 2 times the control value.
• Respiratory failure: The need for mechanical ventilation for at least 48 h.
• Traumatic brain injury: Glasgow Coma Scale score ≤8, traumatic spinal cord injury, or burn injury covering >35% of the body surface area.
• Sepsis: An acute change in total SOFA score ≥2 points consequent to infection.

Minor Risk Factors for SUP:

• Acute or chronic renal failure: Requiring intermittent or continuous renal replacement therapy.
• Shock: Continuous infusion with vasopressors or inotropes, mean arterial blood pressure below 70 mmHg, or plasma lactate level ≥4 mmol/L.
• Chronic hepatic failure: Defined as cirrhosis confirmed by biopsy, with a history of variceal bleeding or hepatic encephalopathy.
• Glucocorticoid therapy: ≥250 mg hydrocortisone equivalent per day.
• Multiple trauma: Injury severity score >16.

No intervention was made in the patients’ SUP practices in either period.

### Sample size

For the study’s sample size, it was determined that there should be at least 42 patients in each period, based on the literature data that inappropriate SUP use in patient groups is reduced by approximately 30% [10], based on the calculation made on alpha 0.05 and 95% power values. Considering the 15% loss margin, it was decided to include 96 patients in the study, with at least 48 patients each period.

### Definitions

Authors defined significant GI bleeding as bleeding requiring a gastroscopy or blood transfusion upon clinician judgment. C. *difficile* infection was defined as the presence of relevant symptoms with positive fecal toxin and/or polymerase chain reaction in ICU patients after initiation of SUP in the ICU.

The rate of SUP use in appropriate indications was accepted as the percentage of PPIs used by a patient according to the SPC SUP guideline for the total number of hospital days.

### Outcomes measurement

Adherence rates to SPC SUP guideline and costs of nonadherence were primary outcome measurements.

### Data analysis

The study used descriptive statistics including mean, median, standard deviation, interquartile range (IQR), count, and percentages to show continuous variables’ central tendency and variability. For categorical variables, frequency, and percentages were given. The Kolmogorov-Smirnov test was used to see if continuous variables followed the normal distribution. The result was non-parametric. The Mann-Whitney U tests were used to compare continuous variables between two groups. Categorical data was compared using Chi-square tests. Risk factors associated with inappropriate SUP use were compared among categorical data. Non-categorical data (e.g., length of stay in the intensive care unit) were categorized as lower and upper values of the median values and risk analysis was performed. Risk values were expressed as odds ratio (OR) and 95% confidence interval (CI) and a p-value less than 0.05 was considered statistically significant. Analysis of the dataset was done on an overall basis with the help of IBM SPSS Statistics for Windows, Version 29.0 (Armonk, New York: IBM Corp.).

### Cost savings analysis

This study compared the costs of SUP agents prescribed for inappropriate indications between PreEd and PostEd. Finally, the SUP cost per patient was determined by multiplying the number of appropriate and inappropriate days of use in both PreEd and PostEd by the cost of PPI. Differences in the SUP costs between PreEd and PostEd are called cost-saving.

The costs for the SUP agents were estimated using current drug prices available from the hospital where this study was conducted. Thus, ten pantoprazole intravenous (IV) ampules were accepted for $3. Only the costs related to PPIs have been calculated. The calculation excluded nursing services and medical supplies.

## Results

**FIGURE 1:**
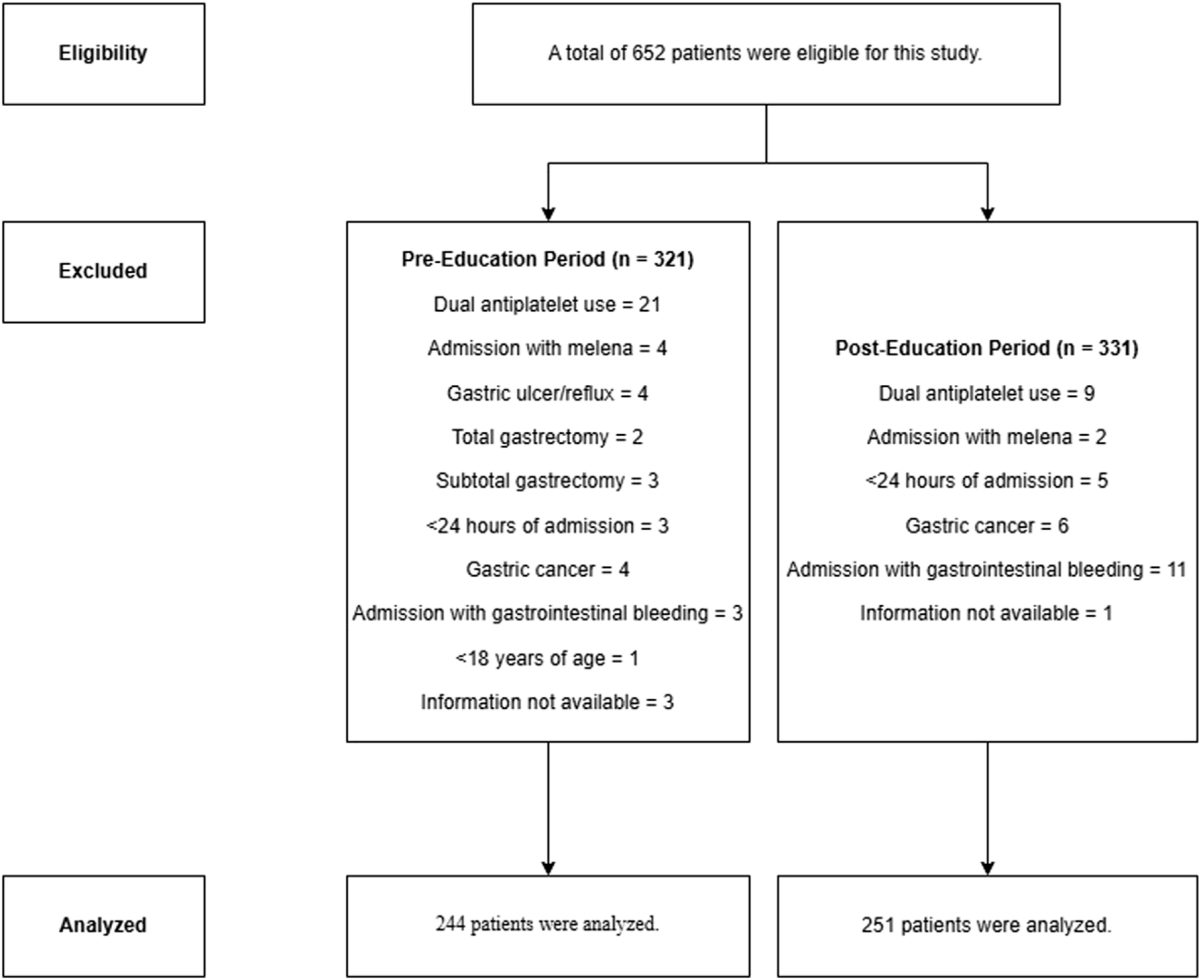
Flow chart of the study.

Six hundred and fifty-two patients were eligible for the study, but 77 PreEd and 80 PostEd patients were excluded due to exclusion criteria. A total of 495 patients were included in the study, 244 in PreEd and 251 in PostEd (Figure 1). The median age (IQR) of all patients was 66 (53–76) years, and 288 (58.2%) were male. ICU admission for surgical reasons was more common in PreEd (45.1%) than in PostEd (35.9%) (p = 0.044). Hypertension at 40.6% and diabetes mellitus at 26.8% are common comorbidities. Other sociodemographic information is shown in Table 1.

**TABLE 1:** Sociodemographic and clinical information of all patients.

Variable	Total (n = 495)	Pre-education (n = 244)	Post-education (n = 251)	*p* value
Age, median (IQR)	66 (53–76)	66.5 (53.25–77.00)	66 (53–76)	0.801
Sex, n (%)				0.927
Female	207 (41.8)	103 (42.2)	104 (41.4)	
Male	288 (58.2)	141 (57.8)	147 (58.6)	
Reason for ICU admission, n (%)				0.044
Surgical	200 (40.4)	110 (45.1)	90 (35.9)	
Medical	295 (59.6)	134 (54.9)	161 (64.1)	
Comorbidities, n (%)				-
Hypertension	201 (40.6)	101 (41.3)	100 (39.6)	
Diabetes mellitus	133 (26.8)	65 (26.6)	68 (27.0)	
Chronic obstructive pulmonary disease	84 (16.9)	44 (18)	40 (15.9)	
Chronic kidney disease	59 (11.9)	26 (10.6)	33 (13.1)	
Cerebrovascular accident	41 (8.2)	17 (6.9)	24 (9.5)	
Asthma	25 (5)	13 (5.3)	12 (4.7)	
Coronary artery disease	83 (16.7)	44 (18)	39 (15.5)	
Heart failure	32 (6.4)	15 (6.1)	17 (6.7)	
Atrial fibrillation	35 (7)	14 (5.7)	21 (8.3)	
Renal status on ICU admission, n (%)				0.08
Normal (eGFR > 60 mL/min/1.73 m^2^)	382 (77.1)	180 (73.7)	202 (80.5)	
Acute kidney failure	51 (10.3)	26 (10.6)	25 (10)	
Chronic renal failure	62 (12.5)	38 (15.7)	24 (9.6)	
CRRT status, n (%)				0.865
Yes	37 (7.5)	19 (9.9)	18 (7.2)	
No	458 (92.5)	225 (90.1)	233 (92.8)	
Total ICU hospitalization (days), median (IQR)	5 (2–12)	5 (2–11)	5 (2–13)	0.457
Discharge status, n (%)				0.205
Transfer to service	343 (69.3)	176 (72.1)	157 (66.5)	
Death	152 (30.7)	68 (37.9)	84 (33.5)	
Oxygen support status, n (%)				0.721
MV	198 (40)	96 (39.3)	102 (40.6)	
Non-invasive MV	78 (15.8)	43 (17.6)	35 (13.9)	
Nasal oxygen supply	58 (11.7)	27 (11.1)	31 (12.4)	
None	161 (32.5)	78 (32)	83 (33.1)	
MV duration (days), median (IQR)	8 (3–18)	7 (3–17)	8.5 (3–19.75)	0.517

CRRT, Continuous renal replacement therapy; ICU, Intensive care unit; IQR, Interquartile range; MV, Mechanical ventilation.

According to the SPC SUP guideline, the mean ± SD rate of patients with appropriate indication was 38.3 ± 41.6% in PreEd and 47.8 ± 42.8% in PostEd (p = 0.005). In both periods, MV ≥ 48 h (35.3%) and coagulopathy (35.1%) were the most common SUP appropriateness criteria. No complications of C. *difficile* infection and gastrointestinal bleeding were observed in patients in both periods. Intravenous pantoprazole was used as the SUP agent in all patients. The total costs of inappropriate indication and PPI use in PreEd and PostEd were 272 dollars and 246 dollars, respectively (p = 0.007). Accordingly, when inappropriate SUP agent use was calculated per patient in both periods, the total cost saving was 34 dollars. Detailed information regarding SUP use, SPC guideline adherence, and associated costs are shown in Table 2.

**TABLE 2:** Data on stress ulcer prophylaxis use and guideline adherence.

	Total (n = 495)	Pre-education (n = 244)	Post-education (n = 251)	*p* value
SUP indication, n (%)				*0.209*
Yes	252 (50.9)	117 (47.9)	135 (53.8)	
No	243 (49.1)	127 (52.1)	116 (46.2)	
SUP usage rate in appropriate indications, mean ± SD (%)	43.1 ± 42.5	38.3 ± 41.6	47.8 ± 42.8	0.005
Number of days for appropriate indication, mean ± SD	6.5 ± 12.8	5.8 ± 12.3	7.3 ± 13.2	0.031
Number of SUP indications, n (%)				-
1	99 (38.6)	51 (21.3)	48 (19.1)	
2	67 (26.1)	29 (11.9)	38 (15.1)	
3	63 (24.6)	31 (12.7)	32 (12.7)	
4	8 (3.1)	6 (2.5)	2 (0.3)	
>4	19 (7.4)	0 (0)	19 (6.3)	
SUP indications^a^, n (%)				-
Major criteria				
Coagulopathy	174 (35.1)	84 (34.1)	90 (35.8)	
≥48 h MV	175 (35.3)	85 (34.5)	90 (35.8)	
Traumatic brain injury	1 (0.2)	1 (0.4)	0 (0)	
Sepsis	121 (24.4)	44 (17.8)	77 (30.6)	
Minor criteria				
Acute or chronic kidney failure	19 (3.8)	16 (6.5)	3 (1.2)	
Septic shock	16 (3.2)	0 (0)	16 (6.3)	
Glucocorticoid use	30 (6)	16 (6.5)	14 (5.5)	
Multiple trauma	2 (0.4)	0 (0)	2 (0.8)	
Chronic hepatic failure	0 (0)	0 (0)	0 (0)	
PPI complications, n (%)				-
Aspiration pneumonia	0 (0)	0 (0)	0 (0)	
Clostridioides difficile infection	0 (0)	0 (0)	0 (0)	
Gastrointestinal bleeding	0 (0)	0 (0)	0 (0)	
SUP cost, mean ± SD ($)				
Inappropriate	1 ± 1.4	1 ± 1.4	0.9 ± 1.4	0.007
Appropriate	1.9 ± 3.8	1.7 ± 3.7	2.1 ± 3.9	0.031
SUP cost, Total ($)				
Inappropriate	518	272	246	0.007
Appropriate	978	427	551	0.031

^a^ Patients have more than one indication for stress ulcer prophylaxis.

MV, Mechanical ventilation; PPI, Proton pump inhibitor; SD, Standart deviation; SUP, Stress ulcer prophylaxis.

Independent clinical factors influenced adherence to the SPC SUP guidelines for appropriate SUP in ICU patients. ICU admission for medical reasons, ICU stays longer than 5 days, ICU stays resulting in death, presence of MV and continuous renal replacement therapy were identified as factors increasing the use of SUP in guideline-compliant indications (OR (95% CI), p; 1.96 (1.35–2.82), <0.001; 0.147 (0.097–0.222), <0.001; 0.020 (0.007–0.055), <0.001; 20.74 (11.65–36.91), <0.001; 6.28 (2.19–18), <0.001, respectively). Age and gender did not affect SUP indication adherence (p > 0.05) (Tables 3, 4).

**TABLE 3:** Statistical analysis of guideline appropriateness of stress ulcer prophylaxis in all patients.

Variable	Indication		p value
	Appropriate (n = 293)	Inappropriate (n = 202)	
Group, n			0.017
Pre-education	131 (44.7)	113 (55.9)	
Post-education	162 (55.3)	89 (44.1)	
Age, median (IQR)	66 (56–78)	65 (50.75–75)	0.079
Sex, n (%)			0.424
Female	121 (44.3)	86 (42.6)	
Male	172 (58.7)	116 (57.4)	
Total ICU hospitalization (days), median (IQR)	9 (4–17)	3 (1–5)	<0.001
Reason for ICU admission, n (%)			<0.001
Surgical	99 (33.8)	101 (50)	
Medical	194 (68.2)	101 (50)	
Discharged status, n (%)			<0.001
Discharged	145 (49.5)	198 (98)	
Death	148 (50.5)	4 (2)	
Renal status on ICU admission, n (%)			<0.001
Normal (eGFR > 60 mL/min/1.73 m^2^)	201 (68.6)	182 (90.1)	
Acute kidney failure + Chronic renal failure	92 (31.4)	20 (9.9)	
CRRT status, n (%)			<0.001
Yes	33 (11.3)	4 (2)	
No	260 (88.7)	198 (98)	
MV status, n (%)			<0.001
Yes	183 (62.5)	15 (7.4)	
No	110 (37.5)	187 (92.6)	
MV duration (days), median (IQR)	9 (4–19)	1 (1–1.25)	<0.001

CRRT, Continuous renal replacement therapy; eGFR, Estimated glomerular filtration rate; ICU, Intensive care unit; IQR, Interquartile range; MV, Mechanical ventillation.

**TABLE 4:** Analysis of relative risk factors for stress ulcer prophylaxis in appropriate indication in all patients.

Risk factors	OR (95% CI)	*p* value
ICU admission for medical reasons	1.96 (1.35–2.82)	<0.001
>5 days ICU stay	0.147 (0.097–0.222)	<0.001
Death	0.020 (0.007–0.055)	<0.001
Mechanical ventillation	20.74 (11.65–36.91)	<0.001
Continuous Renal Replacement Therapy	6.28 (2.19–18)	<0.001

CI, Confidence interval; ICU, Intensive care unit; OR, Odds ratio.

## Discussion

In this study, we assessed the use of SUP, determined the costs of inappropriate use, and highlighted the impact of clinical pharmacists on improving adherence to SUP guideline. It is well documented that SUP is prescribed frequently in the ICU to decrease the incidence of Gİ bleeding. Different studies have revealed that SUP utilization in ICUs is between 81.2% and 92.9% [13–16].

Published literature has varied the rates of adherence to SUP prescriptions in ICUs, thereby showing changes in procedures and guidelines. Various studies have previously reported the rates for inappropriate SUP prescriptions that do not fall within the criteria set in the guidelines as 58–68.1% [5, 13–15, 17, 18]. In contrast to our study, which found 49.1% inappropriate SUP prescriptions, other studies indicated a lower rate of inappropriate SUP prescriptions, ranging from 14% to 38.5% [5, 10, 12, 16]. It was common practice in the center where this study was conducted to prescribe PPIs for SUP to every patient admitted to the ICU at a rate higher than that documented in the literature. However, in this study, the adherence rate according to the SPC guideline was within the range reported in the literature [5, 13–15, 17, 18]. Several reasons contributed to the different rates of inappropriate SUP use in this study compared to others. These factors include the type of hospital, disparities between admissions of medical and surgical patients in the ICU, and assessment of appropriateness by different guidelines and protocols [10, 12, 19]. Thus, based on these studies, one assumes a widespread problem of excessive SUP prescription in ICUs [13, 20–22].

Many studies emphasize the collaborative role of clinical pharmacists in better adherence to SUP guidelines through active management. According to the literature, clinical pharmacists are essential and efficient in prescribing SUP. Critical issues raised include pharmacists’ involvement in visits to the patient, conducting education programs, and making decisions with physicians to optimize SUP practice. Hammond et al. illustrated the powerful positive influence of pharmacist-physician collaboration in the ICU to improve adherence to SUP prescribing guidelines through a structured educational intervention. The authors pointed out that this cost-effective measure easily could have been extrapolated to facilities where pharmacists participate in rounds with physicians [23]. Mahmoudi et al. evaluated the appropriateness of SUP by applying ASHP criteria and studied the economic effects of clinical pharmacist interventions. Their study revealed a significant cost-saving of more than $18,000 monthly from clinical pharmacists’ interventions [16]. Rafinazari et al. did a similar study wherein they concluded that educating physicians about the proper implementation of standard protocols and building up collaboration with clinical pharmacists could result in improved prescribing practices of SUP. Consequently, it results in a relative reduction in hospital expenditures and an absolute reduction of hospital costs and adverse drug reactions [5]. Various strategies have been proposed to tackle the inappropriate use of SUPs. Some have resident training as their component, while some pharmacist-based strategies have also been proposed with encouraging results [9, 10, 24].

The study methodology addresses the cost status of intravenous pantoprazole considering the high dollar exchange rate against the Turkish lira. Because the cost-saving computation only includes patients in PostED, the total cost reduction may appear minimal. Although the cost savings varied in most of the studies where the clinical pharmacist was involved in increasing SUP appropriateness by different strategies, this study confirmed that the inclusion of the clinical pharmacist in the team contributed to cost reduction. Some studies aiming at minimizing inappropriate SUP usage may lead to a decline in appropriate use, putting patients at risk of stress ulcers and their significant sequelae. However, adherence to guidelines could prevent unnecessary adverse effects of SUP medications. In the study by Anstey et al., which attempted to implement the SUP protocol, the incidence of C. *difficile* associated with PPIs decreased from one in ten patients in the pre-and post-implementation groups to one in ten patients [20].

Masood et al. noted that, due to their study’s limitations, they could not follow up with patients for GI bleeding or C. *difficile* infections [8]. Although the adherence rate to the SPC guideline for SUP use was statistically higher in the PostEd period, no GI bleeding or C. *difficile* infection was detected in patients in both periods. So, adhering to guideline-based SUP practices lowers costs, frequency of use, and side effects in our study. However, more research is needed to determine the causal relationship between PPI use, GI bleeding, and C. *difficile* infection [25].

Moreover, the literature consists of studies that determine predictors for inappropriate, excessive usage of SUP in the ICU. These predicting factors are age, sex, length of stay in hospital, reason for admission to medical-surgical ICU, and educational status regarding SUP in various studies [14, 18, 25, 26]. Length of hospital stay and the number of comorbidities were identified as risk factors by Issaa et al. [18]. Alsultan et al. did not find a link between SUP use and hospital stay duration; however, Mayet et al. did find that appropriate acid suppression treatment rates increased with longer lengths of stay [27, 28]. Some studies have, moreover, shown that increasing patient age and gender predict inappropriate PPI use [21, 26, 29–31]. However, a more recent study has shown that the appropriateness of PPI treatment in patients is not influenced by gender [27, 28]. Factors indicating poor prognosis, such as the presence of MV, prolonged hospital stay, and hospitalizations resulting in death, provided reasons for the significant probability of prescribing appropriate SUP to the guideline in this study. Patients with an extended ICU stay are sicker in terms of the underlying medical condition, more prone to developing a more significant number of ICU-related complications, and often carry a poor prognosis. As a result, the longer the stay, the more familiar the major and minor criteria for SUP, making it very common and appropriate in this subset of patients. In the present study, it is observed that inappropriate prescription of SUP is more likely to happen in surgical stays. As has been the case in other studies, this study did not find that age and gender significantly affected the appropriateness of SUP. In this regard, some studies suggesting that gender significantly affects SUP use do not have any rational justification [25, 28, 29]. Moreover, inappropriate SUP prescriptions may be influenced by education. Recent studies found more guideline-adherent SUP prescriptions in academic institutions than nonacademic hospitals [32–34].

This study has several limitations. Firstly, it was conducted at a single center where clinical pharmacy services were implemented for the first time. The study aimed to address the frequent and often inappropriate use of PPIs for SUP in the ICU, highlighting these issues to the team and raising awareness. The education was targeted solely at physicians, as they are the only professionals authorized to prescribe medications in Türkiye, leaving out nurses and other healthcare professionals. While the statistically significant reduction in inappropriate SUP use is promising, it is difficult to definitively attribute these improvements solely to the educational intervention, especially considering the widespread and inappropriate use of SUP prior to the intervention. Lastly, the cost analysis did not include nursing services or other associated expenses, as it was challenging to correlate these variables with costs in such a busy setting. Nonetheless, the current analysis offers a meaningful starting point for evaluating the economic impact of education. The notable features of the study include its status as one of the initial studies undertaken on SUP in the ICU in our country and its ability to arrive at a cost-effective conclusion regarding the use of SUP by the guide. The single-center design and relatively short duration of 6 months limit the generalizability of the findings.

## Conclusion

This study, which is focused on local practice, reflects the international problem of excessive and inappropriate SUP use. Converting this challenge requires cooperation between the clinical pharmacist and the physician. This collaboration has lowered PPI use and associated costs for SUP while promoting safe and cost-effective SUP practices by boosting adherence to guideline-based prescriptions.

## Data Availability

The raw data supporting the conclusions of this article will be made available by the authors, without undue reservation.
